# On the electronic structure and hydrogen evolution reaction activity of platinum group metal-based high-entropy-alloy nanoparticles†

**DOI:** 10.1039/d0sc02351e

**Published:** 2020-08-11

**Authors:** Dongshuang Wu, Kohei Kusada, Tomokazu Yamamoto, Takaaki Toriyama, Syo Matsumura, Ibrahima Gueye, Okkyun Seo, Jaemyung Kim, Satoshi Hiroi, Osami Sakata, Shogo Kawaguchi, Yoshiki Kubota, Hiroshi Kitagawa

**Affiliations:** Division of Chemistry, Graduate School of Science, Kyoto University Kitashirakawa-Oiwakecho, Sakyo-ku Kyoto 606-8502 Japan dongshuangwu@kuchem.kyoto-u.ac.jp kusada@kuchem.kyoto-u.ac.jp kitagawa@kuchem.kyoto-u.ac.jp; Department of Applied Quantum Physics and Nuclear Engineering, Kyushu University Motooka 744, Nishi-ku Fukuoka 819-0395 Japan; The Ultramicroscopy Research Center, Kyushu University Motooka 744, Nishi-ku Fukuoka 819-0395 Japan; Synchrotron X-ray Group, Synchrotron X-ray Station at SPring-8, National Institute for Materials Science Kouto, Sayo-cho, Sayo-gun Hyogo 679-5148 Japan; Center for Synchrotron Radiation Research, Japan Synchrotron Radiation Research Institute 670-5198 Japan; Research & Utilization Division, Japan Synchrotron Radiation Research Institute (JASRI), SPring-8 Kouto, Sayo-cho, Sayo-gun Hyogo 679-5198 Japan; Department of Physical Science, Graduate School of Science, Osaka Prefecture University Sakai Osaka 599-8531 Japan

## Abstract

We report the synthesis of high-entropy-alloy (HEA) nanoparticles (NPs) consisting of five platinum group metals (Ru, Rh, Pd, Ir and Pt) through a facile one-pot polyol process. We investigated the electronic structure of HEA NPs using hard X-ray photoelectron spectroscopy, which is the first direct observation of the electronic structure of HEA NPs. Significantly, the HEA NPs possessed a broad valence band spectrum without any obvious peaks. This implies that the HEA NPs have random atomic configurations leading to a variety of local electronic structures. We examined the hydrogen evolution reaction (HER) and observed a remarkably high HER activity on HEA NPs. At an overpotential of 25 mV, the turnover frequencies of HEA NPs were 9.5 and 7.8 times higher than those of a commercial Pt catalyst in 0.05 M H_2_SO_4_ and 1.0 M KOH electrolytes, respectively. Moreover, the HEA NPs showed almost no loss during a cycling test and were much more stable than the commercial Pt catalyst. Our findings on HEA NPs may provide a new paradigm for the design of catalysts.

## Abstract 2

RuRhPdIrPt high-entropy-alloy nanoparticles with a broad and featureless valence band spectrum show high hydrogen evolution reaction activity.

## Graphical abstract

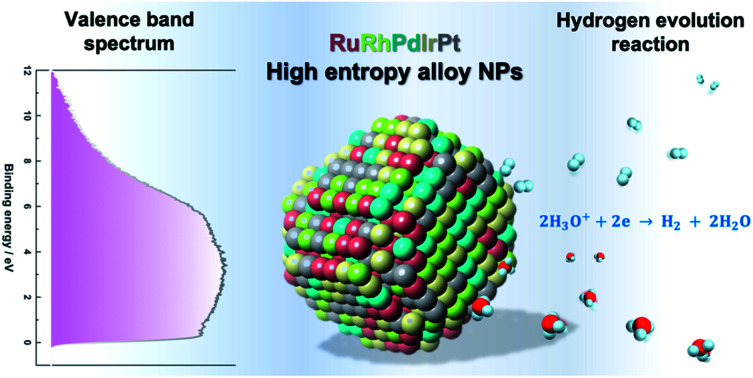

## Introduction

High-entropy alloys (HEAs) are solid solutions consisting of at least five elements in an approximately equal atomic ratio (recently extended to 5 to 35 at%).^1^ Yeh and colleagues have reported that when the number of constituents is more than five, the contribution of configurational entropy (Δ*S* ≥ 1.6 *R*) to the total free Gibbs energy (Δ*G* = Δ*H* − *T*Δ*S*) would overcome the contribution of mixing enthalpy, resulting in the formation of solid-solution rather than the intermetallic phase.^1–3^ Some HEAs are reported to show a balance of high hardness and strength and to have high thermal/chemical stabilities towards oxidation and corrosion.^4,5^ Furthermore, HEAs can provide unique surfaces and electronic structures compared to conventional monometals and binary alloys. Even considering the nearest neighbouring atomic arrangement of one atom in fcc(111) of a quinary alloy, there are more than 10^5^ (5^9^/3 > 10^5^, where 5 means five kinds of elements, 9 is nine nearest neighbouring atoms, and 3 means three-fold symmetry of the fcc(111)) ways, that is, all atoms are in different configurations and have distinct local electronic structures.

It is well known that the catalytic properties of monometals and conventional binary alloys can be understood and even predicted in terms of the electronic structures of the principal elements both experimentally and theoretically (*i.e.* d-band theory).^6–8^ However, due to the heterogeneity of the surface atoms in HEAs, the catalytic properties of HEAs might be not limited by one principle element anymore and the d-band center theory might not be able to describe the activity of HEAs.^9^ The neighbouring active sites with different local electronic structures in HEAs also provide the possibility for tuning the selectively in complex reactions. For example, Yao *et al.* reported that PdPtRhRuCe HEA nanoparticles (NPs) showed almost 100% selectivity towards ammonia synthesis while the phase-separated alloy only had less than 20% selectivity.^10^ We have found that IrOsPtPdRhRu could catalyse ethanol oxidation with a 12-electron process, although the single metals generally prompt a four-electron process at most.^11^ Löffler *et al.* found that Cr–Mn–Fe–Co–Ni complex solid solution showed at least comparable catalytic activity to Pt in oxygen reduction reaction (ORR), in despite that none of its constituents had good ORR activity.^12^ In addition to HEA, other alternative denominations have also been discussed elsewhere, such as complex solid solutions,^12–14^ multiprincipal element alloys,^15^ polyelemental particles,^16,17^ multi-elemental alloy,^18^ and multimetallic clusters/particles.^19^ These kinds of alloys may present a class of promising ideal catalysts having desired activity, stability and selectivity and thus make a paradigm shift in catalysis research.^20^ These alloy catalysts with appealing performances have been reported recently,^10–12,17–19,21–24^ yet, the interpretation on their properties is still challenging. Quite recently, Rossmeisl and co-authors have made a great step in theoretically predicting ORR^25^ and CO_2_ and CO reduction^26^ properties on HEAs by combing density functional theory calculations and supervised machine learning. Chuhmann and colleagues proposed that the multiple wave-shaped segments of the current–potential ORR curves can be used to interpret the adsorption energy distribution pattern.^13^ However, to our knowledge, experimental studies on the relationship between the electronic structure and the catalytic property of HEA have not been conducted.

To date, the reports on HEA NPs are still scarce compared to HEA bulk, and almost all the syntheses require special techniques such as carbothermal shock,^10,18,19,21^ spark ablation,^27^ sputtering,^12^ and mechanical ball milling.^28^ Here, we show the chemical synthesis of single-phase HEA NPs consisting of Ru, Rh, Pd, Ir, and Pt in a nearly equal molar ratio by a simple one-pot polyol method. We investigated the electronic structure of HEA NPs using hard X-ray photoelectron spectroscopy (HAXPES), which is the first direct observation of the electronic structure of HEA NPs. The observed valence band (VB) spectrum are broad without any peaks which implies that the HEA NPs have random atomic configurations leading to a variety of local electronic structures. The hydrogen evolution reaction (HER) was chosen as a target reaction because of the well-established relationships between the catalytic property and the electronic structures of mono(binary) metal catalysts.^6,29^ The obtained **IrPdPtRhRu HEA NPs** greatly outperformed a commercial Pt/C in both acidic and alkaline solutions, with 9.5 and 7.8 times higher turnover frequency (TOF) values at an overpotential of 25 mV, respectively. Plotting the HER activities as a function of the d-band centre showed that the HEA NPs with a superior HER activity did not follow conventional d-band centre theory as expected. In addition, we note that **IrPdPtRhRu HEA NPs** showed much improved catalytic stability than the commercial Pt/C during cycling testing.

## Results and discussion

### Synthesis and characterization of **IrPdPtRhRu HEA NPs**

**IrPdPtRhRu HEA NPs** were obtained by a facile one-pot polyol process. The five metal precursors (in an equimolar ratio) were dissolved in H_2_O, and the aqueous solution was added to the preheated triethylene glycol solution containing poly(*N*-vinyl-2-pyrrolidone) (PVP; K30, MW ≈ 40 000) as a protecting agent at 230 °C. The resulting black solution was cooled to RT and washed several times with a mixture of ethanol, water, and ether to remove the excess PVP. Finally, the black powder was collected by centrifugation and vacuum drying under RT.

The **IrPdPtRhRu HEA NPs** were first characterized by transmission electron microscopy (TEM) and high-angle annular dark-field scanning TEM (HAADF-STEM) coupled with energy-dispersive X-ray spectroscopy (EDX). The bright-field TEM image (Fig. S1a†) shows that the **IrPdPtRhRu HEA NPs** had a mean diameter of 5.5 ± 1.2 nm. The atomic-resolution STEM image of an **IrPdPtRhRu HEA NP** displayed two sets of perpendicular lattice fringes with an equal planar distance of 0.194 nm that can be assigned as the {200} facets of a face-centred cubic (fcc) structure (Fig. S2†).

The structure of **IrPdPtRhRu HEA NPs** was further revealed by synchrotron powder X-ray diffraction (XRD) at the beamline BL02B2, SPring-8.^30^ The Rietveld refinement of the XRD pattern revealed that **IrPdPtRhRu HEA NPs** had an fcc structure with a lattice parameter of *a* = 3.856(1) Å and crystal size of 4.7 nm (Fig. S3†), which are consistent with the STEM results.

As references for catalytic measurements, monometallic Ru, Rh, Pd, Ir, and Pt NPs were also prepared using a polyol method. The diameters of these monometallic NPs were in the range of 2–6 nm (Fig. S1 and S2†).

**Fig. 1:**
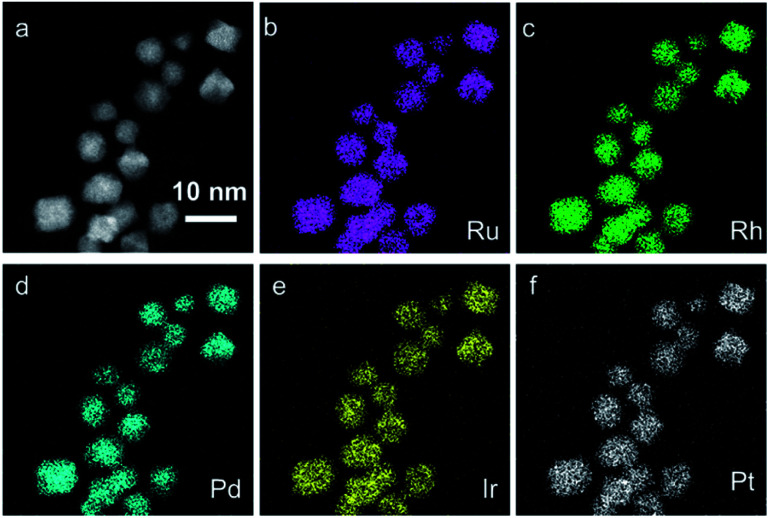
(a) HAADF-STEM image of **IrPdPtRhRu HEA NPs** and its corresponding EDX maps showing (b) Ru-*L*, (c) Rh-*L*, (d) Pd-*L*, (c) Ir-*L*, and (f) Pt-*L*.

**Fig. 2:**
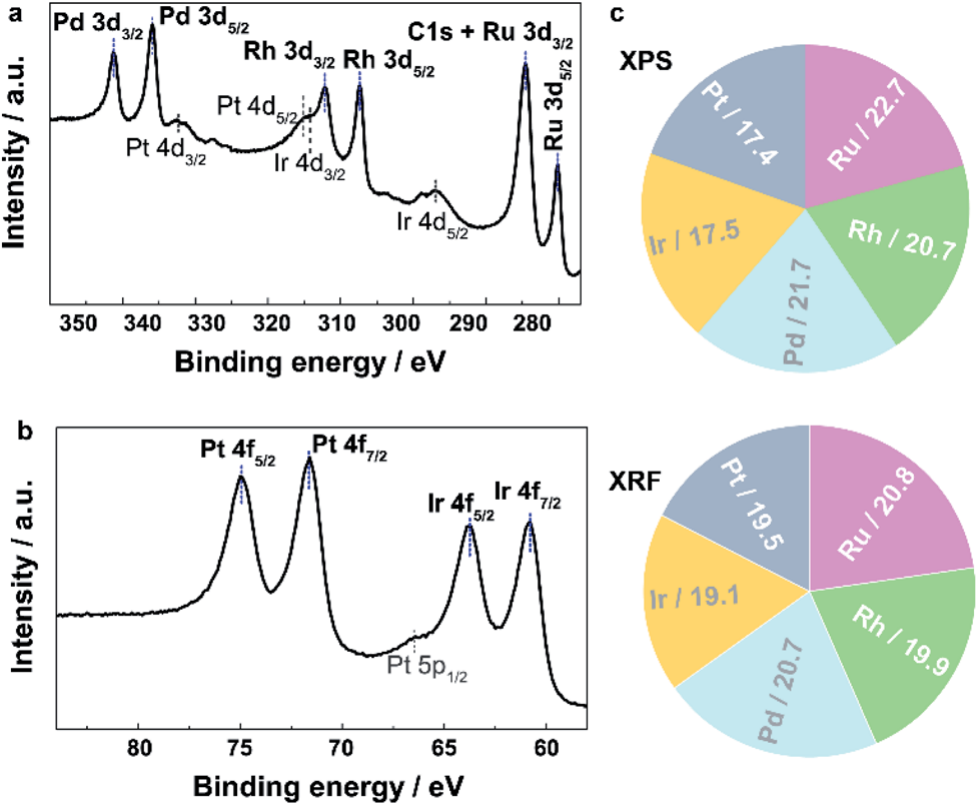
Core-level XPS spectra showing the existence of five elements of **IrPdPtRhRu HEA NPs** in the regions of (a) 277–350 eV and (b) 55–88 eV. The dashed lines assign the peaks. (c) Metallic compositions obtained by XRF and XPS.

To investigate the local structure and compositions in the obtained **IrPdPtRhRu HEA NPs**, we performed electron microscopy, X-ray fluorescence (XRF), and X-ray photoelectron spectroscopy (XPS). Fig. 1 and S4† (a higher resolution) show the HAADF-STEM images and the corresponding EDX maps of Ru, Rh, Pd, Ir and Pt. These maps directly show that each element is homogeneously distributed in the whole NPs, that is, IrPdPtRhRu solid-solution alloys were obtained. XRF measurement revealed that the atomic percentage of each element in **IrPdPtRhRu HEA NPs** was approximately 20% (Fig. 2c and Table S1†). The electronic states were investigated by XPS. As shown in Fig. 2a, in the binding energy range of 280–350 eV, the core-level spectra of Ru 3d, Rh 3d, and Pt 3d were observed as the main peaks with high intensities (blue dashed lines), and Pt 4d and Ir 4d spectra also appeared with low intensities (black dashed lines). In the binding energy range of 60–80 eV, the characteristic doublets from Ir 4f and Pt 4f are seen (Fig. 2b, blue dashed lines). The XPS spectrum of each element was fitted with one component having binding energy similar to its bulk metal^31^ (Fig. S5†), which suggests that the elements in the **IrPdPtRhRu HEA NPs** are in a metallic state. Furthermore, considering the source energy of Mg Kα, the XPS has a probe depth of approximately 1 nm. Therefore, the surface composition of NPs can be investigated by integrating their corresponding XPS peaks. As shown in Fig. 2c and Table S1,† the surface compositions obtained by XPS are similar to the bulk compositions given by XRF.

**Fig. 3:**
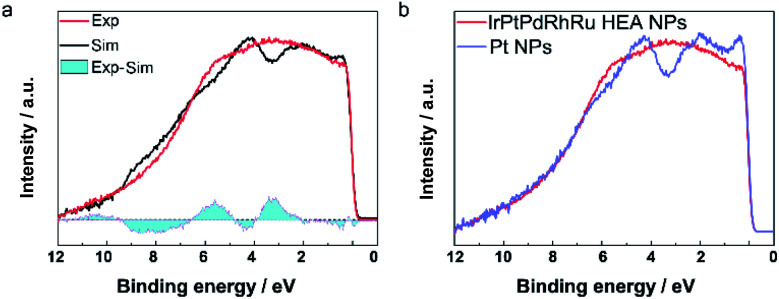
(a) Comparison of the experimental VB spectrum of **IrPdPtRhRu HEA NPs** and the linear combination of VB spectra of the monometal NPs. The difference of the spectra is shown as cyan regions. (b) Comparison of the VB spectra of **IrPdPtRhRu HEA NPs** and Pt NPs.

The catalytic properties are closely related to the valence band (VB) structure of the catalysts; therefore, we performed HAXPES to reveal the VB features of HEA NPs for the first time. We compared the experimental VB spectrum of **IrPdPtRhRu HEA NPs** with the linear combination of the VB spectra of the monometal NPs, which corresponds to a physical mixture of the five elements. As shown in Fig. 3a, obvious differences between these spectra are seen in the cyan regions, which suggests the hybridization of the orbitals of the constituents in **IrPdPtRhRu HEA NPs**. In other words, the difference is derived from the formation of solid-solution NPs, which is consistent with the STEM analyses. We further compared the VB spectrum of **IrPdPtRhRu HEA NPs** with the monometallic NPs in detail. Almost all the monometallic NPs showed obvious peaks in the VB spectra (Fig. 3b and S6†). In contrast, **IrPdPtRhRu HEA NPs** showed a broad VB spectrum without any obvious peaks. We calculated and summarized the d-band centre of each catalyst from the VB spectrum (Table S2†). There was some deviation between our results and the reported theoretical values,^6^ which may originate from the difference between NPs and simple slab models. The d-band centre of **IrPdPtRhRu HEA NPs** was located between those of Pt and Ir NPs.

### Catalytic performance of HER

To investigate the HER performance of **IrPdPtRhRu HEA NPs**, we first loaded the as-prepared NPs onto active carbon (Vulcan XC-72R) with a weight percentage of metal at around 20%. A catalyst ink with a metal concentration of 0.5 mg mL^−1^ was prepared by mixing the carbon-loaded NPs with isopropanol, water, and Nafion. Then, 0.01 mL of the ink was dropped on a rotating disk electrode and dried at RT. For comparison, commercial Pt/C or monometallic Ru, Rh, Pd, Ir, or Pt NPs electrodes were prepared by the same method.

**Fig. 4:**
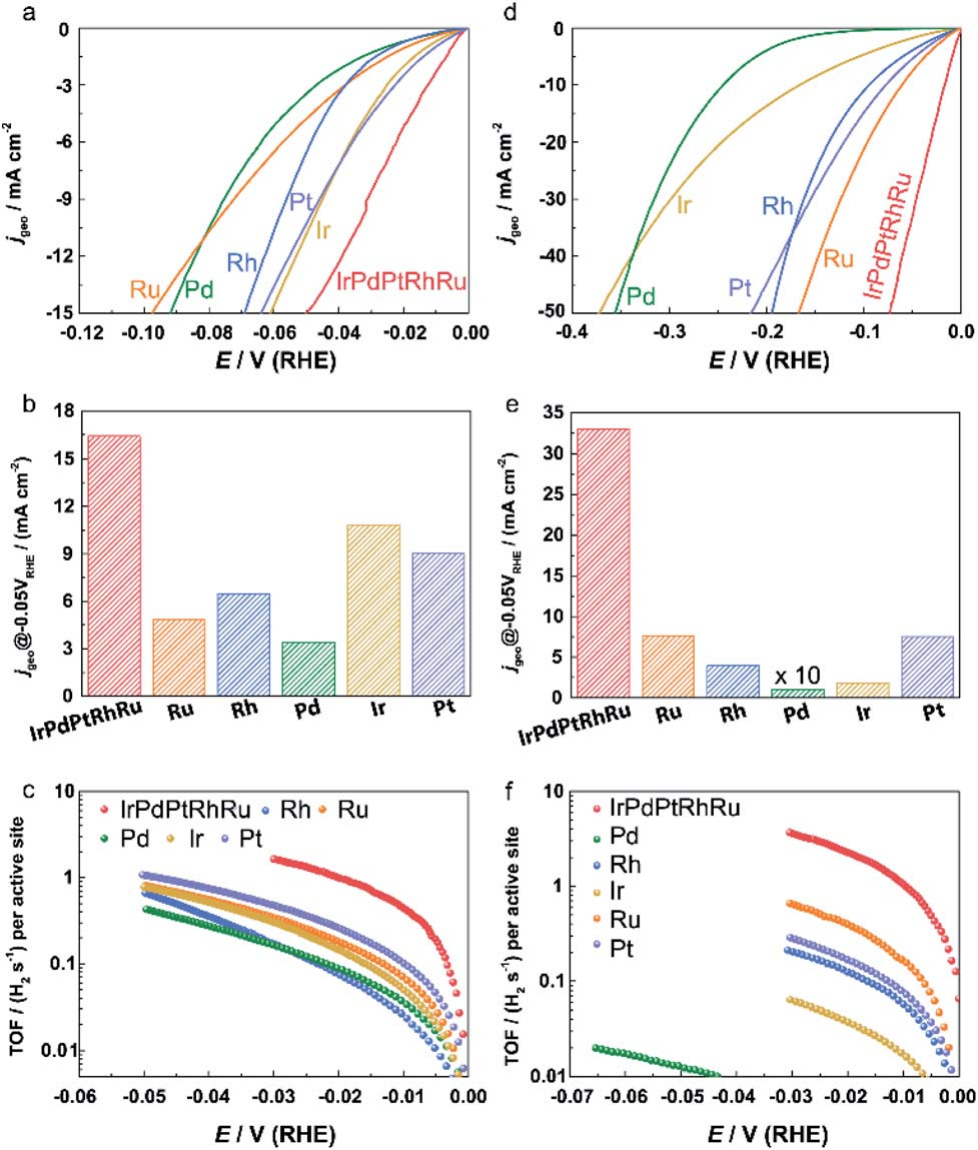
(a and d) Polarization curves, (b and e) geometric current densities at an overpotential of 50 mV, and (c and f) TOF of **IrPdPtRhRu HEA NPs** and other monometallic catalysts in (a–c) 0.05 M H_2_SO_4_ solution and (d–f) 1.0 M KOH solution.

The HER activities of **IrPdPtRhRu HEA NPs** were investigated in both acidic and alkaline electrolytes. The polarization curves obtained at a scan rate of 5 mV s^−1^ under a rotation speed of 1600 rpm in 0.05 M H_2_SO_4_ and 1.0 M KOH are shown in Fig. 4a and c, respectively. The current densities are normalized by the geometric electrode area (0.196 cm^2^, marked as *j*_geo_). At an overpotential of 50 mV, the *j*_geo_ of **IrPdPtRhRu HEA NPs** is 3.4 (4.3), 2.5 (8.4), 4.8 (321.5), 1.5 (18.3), and 1.6 (15.2) times higher than those of the monometallic Ru, Rh, Pd, Ir, and Pt catalysts in acidic (alkaline) solution, respectively (Fig. 4b and e). On an overpotential basis, **IrPdPtRhRu HEA NPs** require much lower overpotentials (33.0/17.0 mV) to achieve a 10 mA cm_geo_^−2^ than the monometallic Ru (77.1/60.4 mV), Rh (58.6/94.6 mV), Pd (78.4/245.0 mV), Ir (47.8/166.0 mV), and Pt (48.9/77.4 mV) catalysts in acidic/alkaline electrolytes (Fig. S7†).

TOF is another important figure of merit used to reveal the intrinsic electrocatalytic activity. The TOF values for each active site of these catalysts were based on the evaluation of the numbers of active sites using a Cu underpotential deposition method that could be adapted for all platinum group metals (Fig. S8 and S9†).^32–35^ We note that the catalytic activities of homemade Pt NPs and commercial Pt/C are similar (Fig. S10†). As shown in Fig. 4c and f, the **IrPdPtRhRu HEA NPs** show much higher TOF values over the whole potential ranges than the monometallic catalysts. The TOF values at an overpotential of 25 and 50 mV of **IrPdPtRhRu HEA NPs** were 1.4 and 3.2 H_2_ per s in 0.05 M H_2_SO_4_, respectively, which were 7.8 and 3.1 times higher than those of the commercial Pt/C catalyst (Fig. S9a†) and 2.9–11.2 times higher than those of other monometallic NPs (Fig. 4c). In 1.0 M KOH, the TOF values of **IrPdPtRhRu HEA NPs** were even larger (3.0 H_2_ per s at 25 mV; 6.4 H_2_ per s at 50 mV) than those of Pt/C (0.3 H_2_ per s at 25 mV; 0.8 H_2_ per s at 50 mV) (Fig. S10b†) and the other monometallic catalysts (Fig. 4f). Moreover, we note that these TOF values are also larger than those of reported highly active catalysts tested under similar conditions, such as Ru@C_2_N (1.66 H_2_ per s at 50 mV),^36^ MoS_2_/NF (1.66 H_2_ per s at 50 mV),^37^ and Au–Ru NWs (0.31 H_2_ per s at 50 mV).^38^

**Fig. 5:**
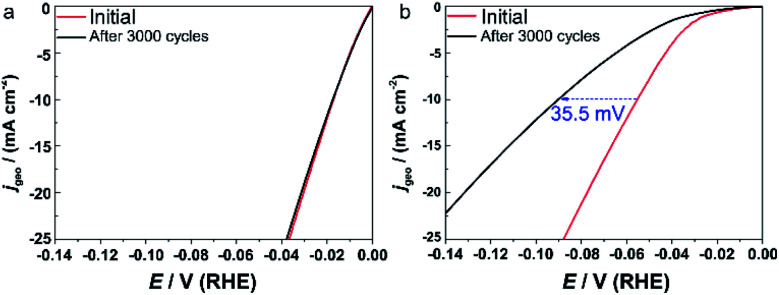
Comparison of the polarization curves between the initial cycle (red) and after 3000 cycles (black). (a) **IrPdPtRhRu HEA NPs** and (b) commercial Pt/C catalyst. The dashed arrow demonstrates the loss of overpotential at a *j*_geo_ of 10 mV cm^−2^.

Because the degradation of commercial Pt/C is more serious in an alkaline solution than an acidic electrolyte,^39^ we focused on the stability in 1.0 M KOH here. The catalysts were subjected to a continuous cyclic voltammetry measurement (3000 cycles, 100 mV s^−1^) into the HER potential region. Fig. 5 shows the comparison of the initial polarization curve with that after 3000 cycles. **IrPdPtRhRu HEA NPs** showed no apparent loss in the current density (Fig. 5a). In contrast, there was a serious loss in the activity of commercial Pt/C, that is, a loss of 35.5 mV in the overpotential at 10 mA cm_geo_^−2^ (Fig. 5b). This result shows the superior stability of **IrPdPtRhRu HEA NPs** compared with the commercial Pt/C catalyst. We suppose that the long cycling life of **IrPdPtRhRu HEA NPs** might be derived from the high corrosion resistance of HEA alloys.^4^

**Fig. 6:**
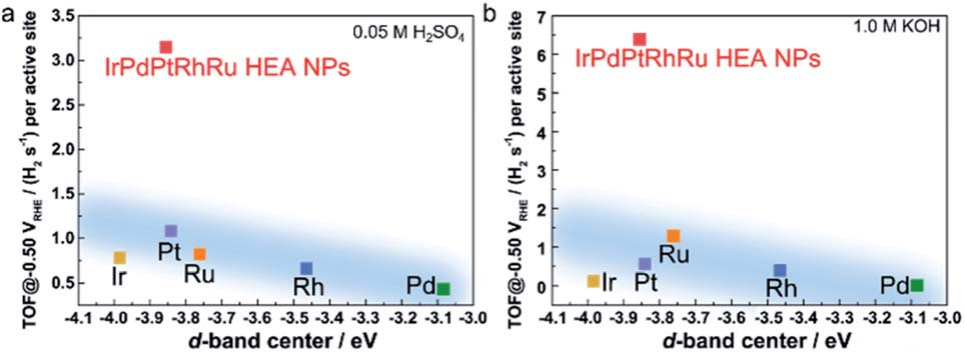
TOF value at −0.05 V_RHE_ as a function of the experimental d-band centre of the tested catalysts in (a) 0.05 M H_2_SO_4_ and (b) 1.0 M KOH solutions. The d-band centre is relative to the Fermi level. The light blue regions show the trend of the activity following d-band centre theory.

It is widely accepted that the intrinsic HER activities of catalysts (such as TOF or exchange current) are mainly determined by the adsorption energy of the intermediate H species.^6,29^ For the transition single metals, the intrinsic HER activity increases the bonding between the metal and the surface H species becomes weaker.^29^ To understand the very high HER performance of **IrPdPtRhRu HEA NPs**, the TOF values at 50 mV were plotted as a function of experimental d-band centre (set Fermi level at zero binding energy) because the metal–H binding of monometallic and most binary catalysts can be inferred from the d-band centre.^11^ When the d-band centre moves to lower energy, the metal–H bonding becomes weaker and thus HER activity increases. As shown in Fig. 6, the TOF of the monometallic NPs is roughly correlated with their d-band centre (light blue region), that is, a deeper d-band centre corresponds to higher activity, which is consistent with previous reports.^29,40^ Here, we note that the Ir NPs showed a slight deviation that might originate from the high percentage of IrO_*x*_ on the surfaces. The **IrPdPtRhRu HEA NP** catalyst has deeper d-band centre locations (between Ir and Pt); thus, it was assumed to intrinsically have similar activity to the Pt or Ir catalyst. The TOF values of **IrPdPtRhRu HEA NPs** in both acidic and alkaline solutions are very high and do not follow d-band centre theory as expected. This suggests that the adsorption energies of H on the active sites of the HEA NP surface during HER cannot be simply estimated from d-band centre theory. These results might originate from the very complex atomic arrangements of the surface and the diverseness of the local density of states of the surface sites, as represented in the HAXPES VB spectrum. Therefore, to understand the reaction mechanism, it would be useful to use a combination of density functional theory calculations and machine learning. For example, Rossmeisl group reported a way to understand the ORR activity of PtPdRhIrRu HEAs on the quantum mechanical level.^25^

## Conclusions

In summary, we prepared **IrPdPtRhRu HEA NPs** by a facile one-pot polyol synthesis for the first time. We experimentally observed the VB spectrum of **IrPdPtRhRu HEA NPs** by HAXPES. The results revealed that HEA NPs have a broad and featureless VB spectrum, thus indicating that HEA NPs contain a variety of different atomic arrangements with unique local density of states. Furthermore, the obtained HEA NPs showed much more enhanced HER activities than the monometallic NPs and commercial Pt/C catalysts in both acidic and alkaline solutions, in addition to high stability. This enhanced activity did not follow the trend of the d-band theory in monometallic catalysts.

Understanding theoretically the relationship between the electronic structure and catalytic properties of HEA NPs remains a challenging issue. However, given the uncountable combinations of HEAs, HEA NPs can be a major future research target in the field of catalysis.

## Authors contribution

D. W., K. K., and H. K. conceived the idea and designed the research. D. W. and K. K. performed the synthesis and general characterization. D. W. performed the electrochemical tests. T. Y., T. T., and S. M. conducted the HAADF-STEM measurements. G. I., O. S., J. K., S. H. and O. S. performed the HAXPES experiments. S. K. and Y. K. contributed to the synchrotron XRD measurements. D. W., K. K., and H. K. discussed the results and wrote the manuscript. All of the authors discussed and commented on the manuscript.

## Conflicts of interest

There are no conflicts to declare.

## Supplementary Material

SC-011-D0SC02351E-s001
