# Correction: On the electronic structure and hydrogen evolution reaction activity of platinum group metal-based high-entropy-alloy nanoparticles

**DOI:** 10.1039/d1sc90104d

**Published:** 2021-05-14

**Authors:** Dongshuang Wu, Kohei Kusada, Tomokazu Yamamoto, Takaaki Toriyama, Syo Matsumura, Ibrahima Gueye, Okkyun Seo, Jaemyung Kim, Satoshi Hiroi, Osami Sakata, Shogo Kawaguchi, Yoshiki Kubota, Hiroshi Kitagawa

**Affiliations:** Division of Chemistry, Graduate School of Science, Kyoto University Kitashirakawa-Oiwakecho, Sakyo-ku Kyoto 606-8502 Japan dongshuangwu@kuchem.kyoto-u.ac.jp kusada@kuchem.kyoto-u.ac.jp kitagawa@kuchem.kyoto-u.ac.jp; Department of Applied Quantum Physics and Nuclear Engineering, Kyushu University Motooka 744, Nishi-ku Fukuoka 819-0395 Japan; The Ultramicroscopy Research Center, Kyushu University Motooka 744, Nishi-ku Fukuoka 819-0395 Japan; Synchrotron X-ray Group, Synchrotron X-ray Station at SPring-8, National Institute for Materials Science Kouto, Sayo-cho, Sayo-gun Hyogo 679-5148 Japan; Center for Synchrotron Radiation Research, Japan Synchrotron Radiation Research Institute 670-5198 Japan; Research & Utilization Division, Japan Synchrotron Radiation Research Institute (JASRI), SPring-8 Kouto, Sayo-cho, Sayo-gun Hyogo 679-5198 Japan; Department of Physical Science, Graduate School of Science, Osaka Prefecture University Sakai Osaka 599-8531 Japan

## Abstract

Correction for ‘On the electronic structure and hydrogen evolution reaction activity of platinum group metal-based high-entropy-alloy nanoparticles’ by Dongshuang Wu *et al.*, *Chem. Sci.*, 2020, **11**, 12731–12736, DOI: 10.1039/D0SC02351E.

The authors regret that there was an error in Fig. 6 of the original article. In the original article, the d-band center values shown in Fig. 6 and Table S2 had been miscalculated. The correct version of Fig. 6 is shown below, and the ESI available online has also been changed to show the correct version of Table S2. The tendency in the activity and d-band center has not changed, and the new data have no influence on the conclusions of the paper.

**Fig. 6:**
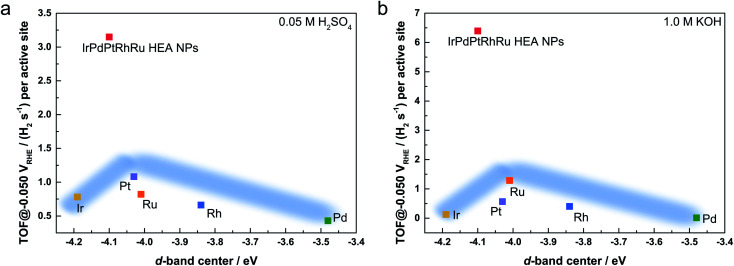
TOF value at −0.05 V_RHE_ as a function of the experimental d-band center of the tested catalysts in (a) 0.05 M H_2_SO_4_ and (b) 1.0 M KOH solutions. The d-band center is relative to the Fermi level. The light blue regions show the trend of the activity following d-band center theory.

The Royal Society of Chemistry apologises for these errors and any consequent inconvenience to authors and readers.

