# Supplementary material for: On the electronic structure and hydrogen evolution reaction activity of platinum group metal-based high-entropy-alloy nanoparticles
Source: Chem Sci. 2020 Aug 11;11(47):12731–6. doi: 10.1039/d0sc02351e (PMC8163215; doi:10.1039/d0sc02351e)
Supplement: SC-011-D0SC02351E-s001 [file SC-011-D0SC02351E-s001.pdf]

Electronic Supplementary Material (ESI) for Chemical Science.
This journal is © The Royal Society of Chemistry 2021

Electronic Supplementary Information

On the Electronic structure and hydrogen evolution reaction activity of platinum group metal-based high-entropy-alloy nanoparticles

Dongshuang Wu, *[a] Kohei Kusada, *[a] Tomokazu Yamamoto,[b] Takaaki Toriyama,[c] Syo Matsumura,[b,c] Gueye Ibrahima,[d] Okkyun Seo,[d] Jaemyung Kim,[d] Satoshi Hiroi,[d] Osami Sakata,[d,e] Shogo Kawaguchi,[f] Yoshiki Kubota [g] and Hiroshi Kitagawa*[a]

[a.] *Division of Chemistry, Graduate School of Science, Kyoto University, Kitashirakawa-Oiwakecho, Sakyo-ku, Kyoto 606-8502, Japan.*

[b.] *Department of Applied Quantum Physics and Nuclear Engineering, Kyushu University, Motooka 744, Nishi-ku, Fukuoka 819-0395, Japan.*

[c.] *The Ultramicroscopy Research Center, Kyushu University, Motooka 744, Nishi-ku, Fukuoka 819-0395, Japan.*

[d.] *Synchrotron X-ray Group and Synchrotron X-ray Station at SPring-8, National Institute for Materials Science, Kouto, Sayo-cho, Sayo-gun, Hyogo 679-5148, Japan.*

[e.] *Center for Synchrotron Radiation Research, Japan Synchrotron Radiation Research Institute, 670-5198, Japan*

[f.] *Research & Utilization Division, Japan Synchrotron Radiation Research Institute (JASRI), SPring-8, Kouto, Sayo-cho, Sayo-gun, Hyogo 679-5198, Japan.*

[g.] *Department of Physical Science, Graduate School of Science, Osaka Prefecture University, Sakai, Osaka 599-8531, Japan.*

Email: dongshuangwu@kuchem.kyoto-u.ac.jp, kusada@kuchem.kyoto-u.ac.jp, Kitagawa@kuchem.kyoto-u.ac.jp

Note added after first publication, 5th May 2021:

1. Page 4, Equation 1, $\frac{\int_{E_1}^{E_2} E \times I(E)d(E)}{v \times 0.42}$ was changed to $\frac{\int_{E_1}^{E_2} I(E)d(E)}{v \times 0.42}$

2. Page 15, Table S2, the d-band center values were corrected.

Synthesis of IrPdPtRhRu HEA NPs and other monometallic NPs

For the synthesis of **IrPdPtRhRu HEA NPs**, five metal precursors, $RuCl_3 \cdot nH_2O$, $RhCl_3 \cdot nH_2O$, K_2PdCl_4, K_2PtCl_4, and H_2IrCl_6 were dissolved in ultra-pure water (0.2 mmol of each precursor, total 50 mL water). Next, the mixture was sprayed into the preheated triethylene glycol (TEG, 300 mL) solution containing dissolved poly(N-vinyl-2-pyrrolidone) (PVP; K30, MW ≈40000, Wako, 1110 mg) at 230 °C. After adding all the mixture, the solution was maintained at 230 °C for 10 min and then cooled to RT. The **IrPdPtRhRu HEA NPs** were separated from the solution by centrifugation with acetone and diethylene ether. After washing the NPs several times by acetone, diethylene ether, ethanol, and water to remove excess PVP, the resultant black powders were vacuum dried under RT. Monometallic NPs were obtained by a similar process by adding the corresponding metal precursors.

Characterization

Low-magnification bright-field TEM images were taken at 100 kV using an HT7700 TEM microscope (Hitachi, Japan). HAADF-STEM and EDX analyses were conducted on a JEM-ARM200F STEM instrument (JEOL, Japan) with an aberration corrector operated at 200 kV. Synchrotron XRD patterns were measured at the BL02B2 beamline, SPring-8, Japan with a radiation wavelength of 0.63 Å. HAXPES measurements were performed by employing a linearly polarized X-ray beam in the horizontal plane with a photon energy of 5.95 keV at the undulator beamline BL15XU of the National Institute for Materials Science at SPring-8 in Japan. Lab XPS was performed on a Shimadzu ESCA-3400 X-ray photoelectron spectrometer (Shimazu, Japan) using a Mg $K\alpha$ anode. XPS spectra were calibrated to the C1s peak at 284.8 eV using carbon paste as the inner reference. XRF (ZSX Primus IV, Rigaku, Japan) was used to measure the atomic ratio of each metal in the as-prepared materials.

Hard X-ray photoelectron spectroscopy (HAXPES)

HAXPES samples were prepared by mixing the sample with carbon powder in a weight ratio of 2:1. The total energy resolution was estimated to be 0.24 eV by measuring the Fermi cut-off of an evaporated Au thin film. The binding energy referred to the Fermi level of the Au film. For the VB spectra analyses, a real background subtraction is performed by measuring the carbon power and PVP VB spectra. All the VB spectra were normalized in the same region (0–12 eV). The *d*-band is obtained by using the following equation:

$$\overline{E}_{d-band} = \frac{\int E \times DOS(E)dE}{\int DOS(E)dE}$$

where *E* is a binding energy and *DOS(E)* is the DOS of the occupied *d* states. Because of the small contribution of the *s* band to the VB spectrum of the *d*-metal, here we used the *I(E)* (intensity of a VB spectrum) to represent the *DOS(E)*, and the centre position is defined as the centre of gravity of the VB spectra.

$$\overline{E}_{d-band}^{obs} = \frac{\int E \times I(E)dE}{\int I(E)dE}$$

The calculated VB spectra are obtained by a linear combination method based on the XRF results.

Electrochemical measurements

All electrochemical measurements were conducted at RT and repeated three times to ensure reproducibility using an electrochemical workstation (CHI760e, CH Instruments, USA). The electrolyte was prepared using concentrated H_2SO_4 or KOH pallets in Millipore water (>18.2 MΩ cm). The pH value of the electrolyte was measured using a pH meter (PH-6011A, CUSTOM Co., Japan) every time before use. The resultant NPs were first loaded on commercial carbon by sonicating the NPs and carbon mixture in an isopropanol and water solution for around 4 h at RT. The weight percentage of the metal on the carbon-loaded catalysts was determined by the elementary analysis with approximately 20% for all the samples. The electrocatalyst inks contained 0.5 mg/mL of metals and were prepared by dispersing the carbon-loaded NPs in a

mixture of isopropanol, water, and 0.05 wt.% Nafion. A Ag/AgCl reference electrode (3 M NaCl) for acidic media and a Hg/HgO reference electrode (1.0 M KOH), a carbon rod electrode, and a rotating disk electrode (RDE, ϕ 5.0 mm) were used during the measurements. The catalyst ink (ca. 10 μL) was dropped onto the RDE surfaces and dried under air overnight.

In a general procedure of HER, the electrode was first subjected to several hundred cycles of voltammogram (CVs) tests with a high scan speed (500 mV/s) in an Ar-saturated electrolyte to obtain a stable surface. The HER was evaluated by a linear scanning voltammogram technique with a rotation speed of 1600 rpm/s and a scan rate of 5 mV/s. All the polarization data were corrected with the solution resistance at a compensation ratio of 80%.

The number of active sites (n) and turnover frequency (TOF) of catalysts were calculated based on the electrochemical active surface area (EASA) measured by a Cu underpotential deposition (UPD) method.[1-4] For measuring EASA, first, the electrodes were cleaned by several hundreds of CVs in the range of 0.05–1.05 V with a high scan speed (500 mV/s) in an Ar-saturated 0.5 M H_2SO_4 solution to obtain a stable surface. Next, we maintained the electrode at a potential of –0.02 V for around 2 min to eliminate the influence of surface oxides. Then, we recorded a fine CV at a scan rate of 10 mV/s. We kept the electrode in the Ar-saturated 0.5 M H_2SO_4 + 0.05 M $CuSO_4$ solution at a certain potential for 100 s and a CV scan with an anodic sweep direction was recorded from the specified potential to the upper limit at a scan rate of 10 mV/s. The specified potential was changed until a bulk deposition of Cu was observed in the CV profile. The EASA was calculated based on integrating the Cu UPD peak area, as seen in Equation (1). The number of n and TOF were calculated based on Equations (2) and (3), respectively:

$$EASA = \frac{Q_{Cu}}{0.42} = \frac{\int_{E_1}^{E_2} I(E)d(E)}{v \times 0.42} \quad (1)$$

$$n = \frac{Q_{Cu}}{2F} \quad (2)$$

$$TOF = \frac{I}{2Fn} \quad (3)$$

where Q_{Cu} is the voltammetry charge of Cu UPD after deducting the black CV profile in pure 0.5 M H_2SO_4. E_1 and E_2 are the lower and upper limits of the Cu UPD peak, respectively, and v is the scan rate of the CV profile. The conversion factor for the two-electron Cu deposition process was 0.42 mC cm^{-2}. F is the Faraday constant (96480 C mol^{-1}).

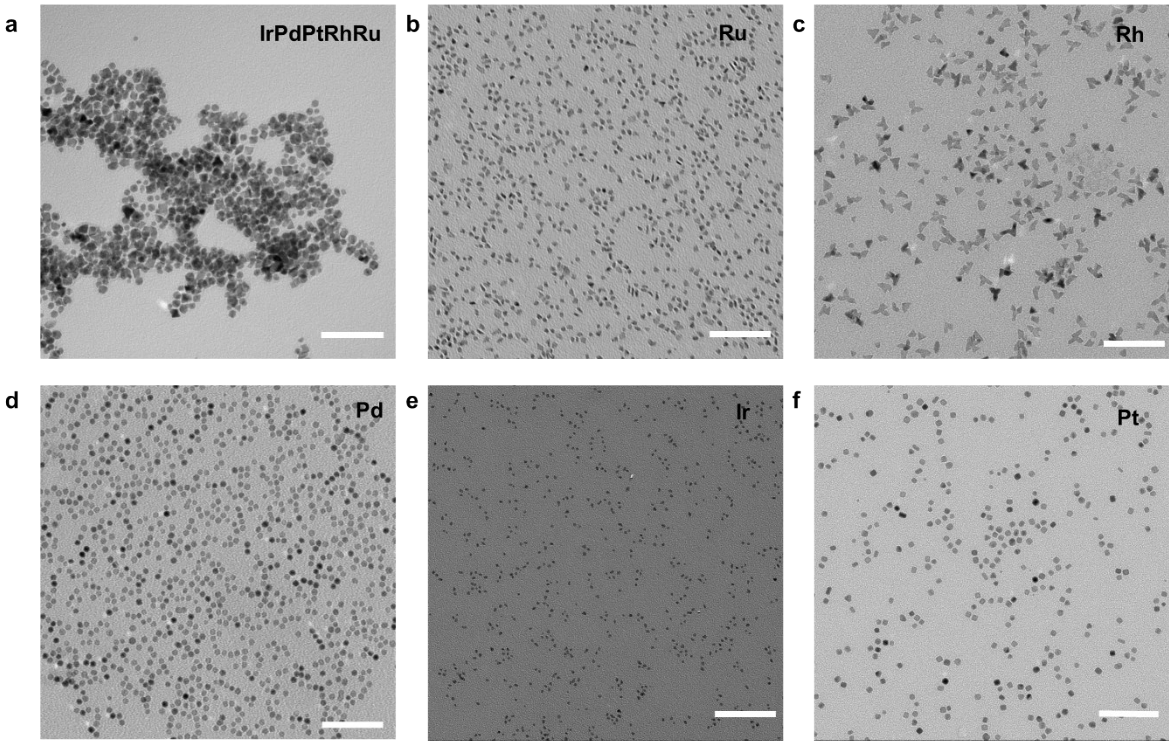

Fig. S1 Bright-field TEM images of (a) **IrPdPtRhRu HEA NPs**, (b) Ru NPs, (c) Rh NPs, (d) Pd NPs, (e) Ir NPs, and (f) Pt NPs. The mean diameters of these NPs were 5.5 ± 1.2, 4.9 ± 0.8, 5.5 ± 1.1, 4.5 ± 0.6, 1.9 ± 0.3, and 4.3 ± 0.6 nm, respectively. The scale bar in each figure is 50 nm.

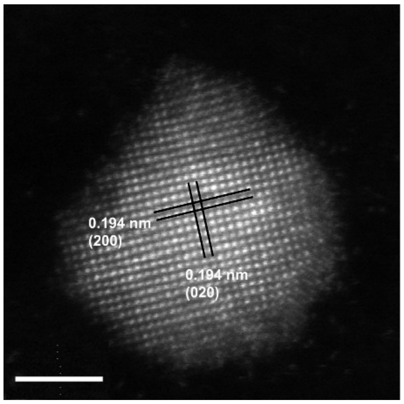

Fig. S2 HAADF-STEM image of an **IrPdPtRhRu HEA NP** showing the perpendicular lattice planes (black lines) with the same planar distance of 0.194 nm that can be assigned as the {200} facets of the fcc structure. The scale bar is 2 nm.

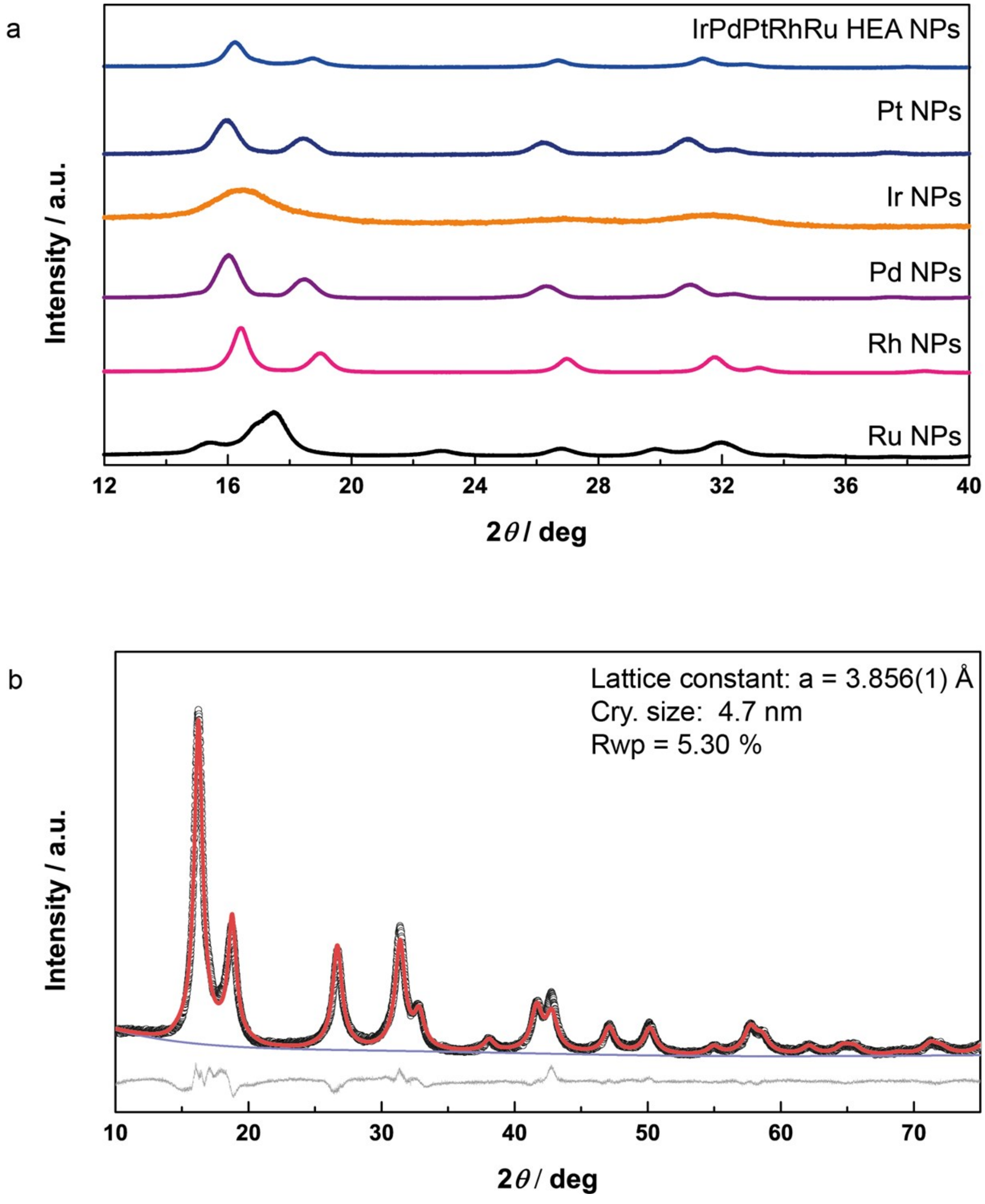

Fig. S3 (a) Synchrotron XRD patterns of **IrPdPtRhRu HEA NPs** and the obtained monometallic NPs in the range of 2θ = 12–40° measured at RT. Wavelength: λ = 0.62938(6) Å. (b) Rietveld refinement in the full range (2θ = 10–70°) of the XRD of **IrPdPtRhRu HEA NPs**. The raw data, fitting profile, differences, and background are shown as black open circles and red, light purple, and grey solid lines, respectively.

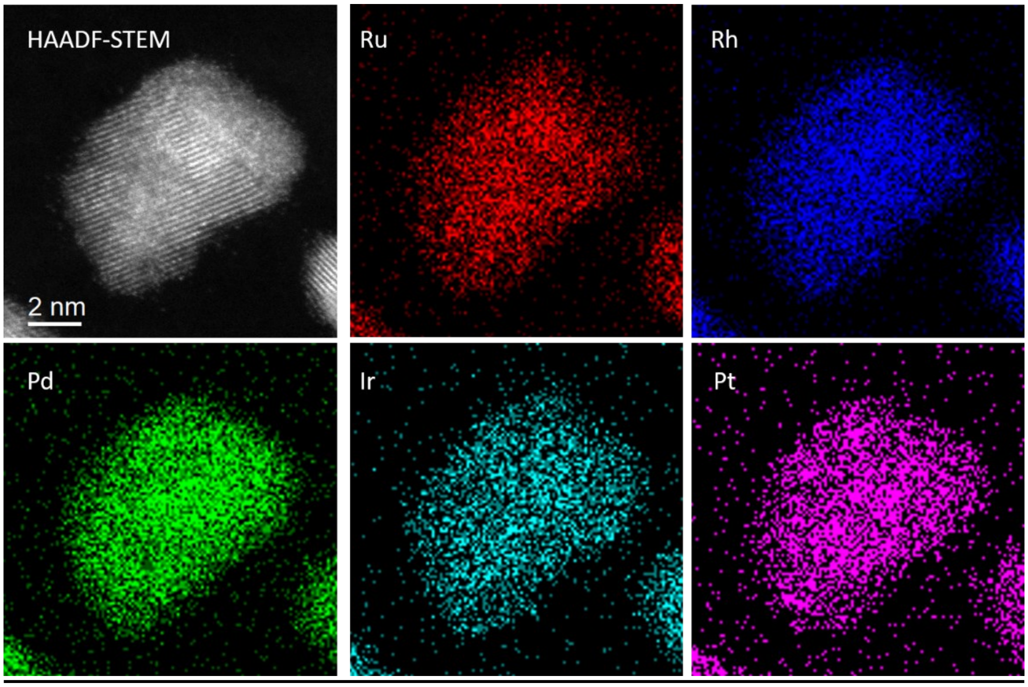

Fig. S4. High-resolution HAADF-STEM image and the corresponding EDX maps showing each constituent.

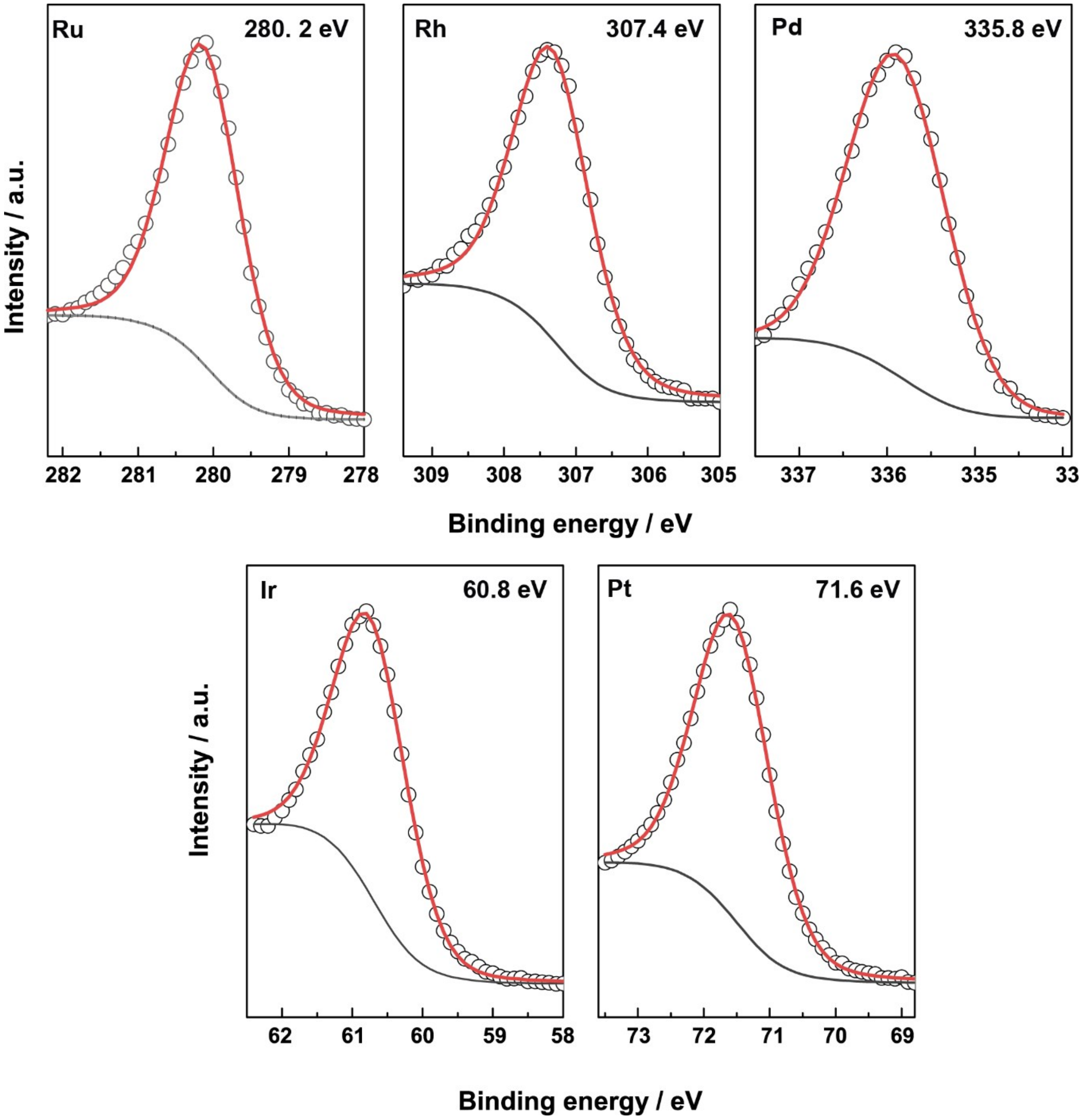

Fig. S5 Experimental (black circles) and fitted (red line) spectra of Ru 3*d*5/2, Rh 3*d*5/2, Pd 3*d*5/2, Ir 4*f*7/2 and Pt 4*f*7/2. The Shirley background is shown by the grey line.

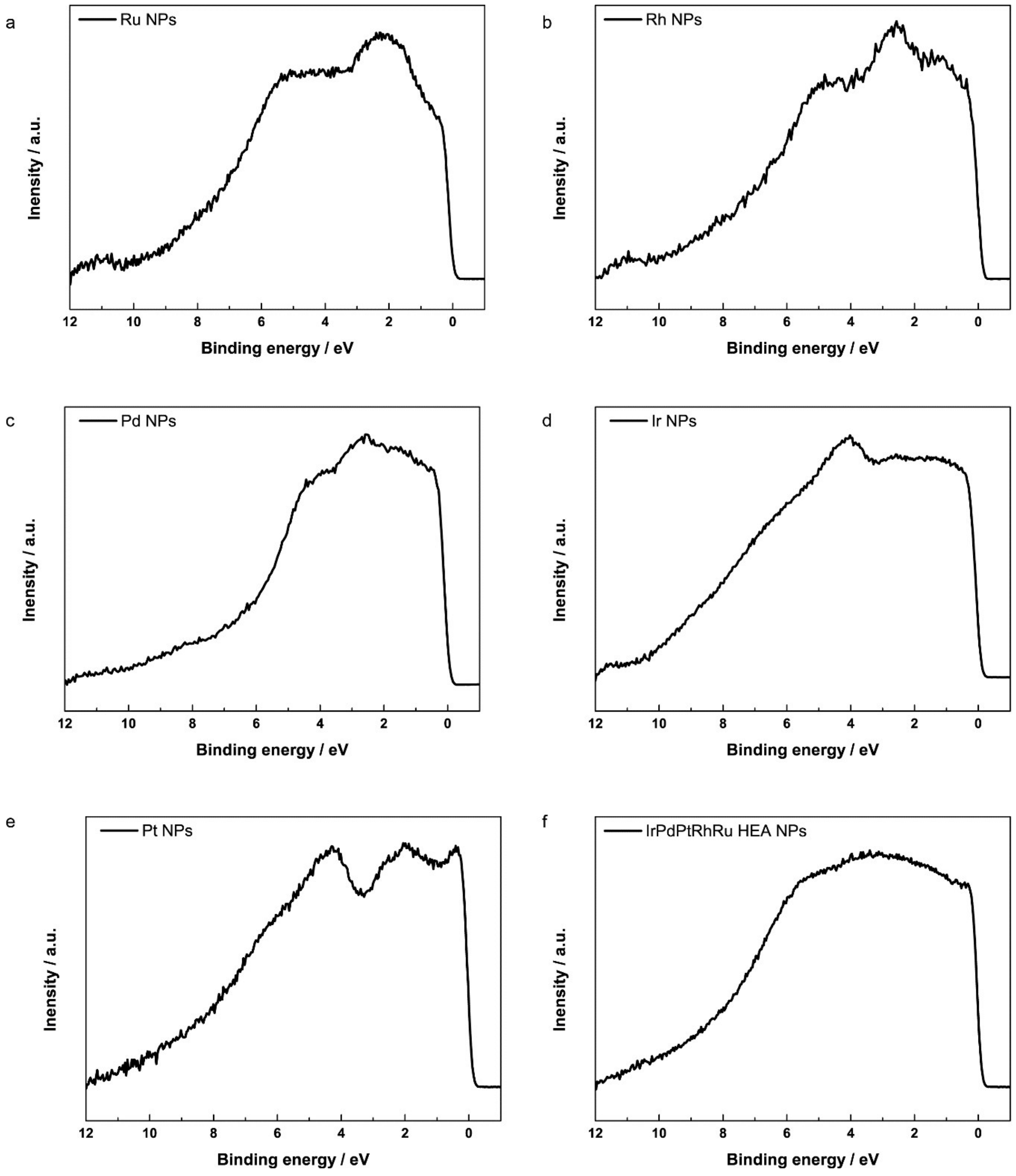

Fig. S6 VB spectra of (a) Ru, (b) Rh, (c) Pd, (d) Ir, (e) Pt, and (f**) IrPdPtRhRu HEA NPs**.

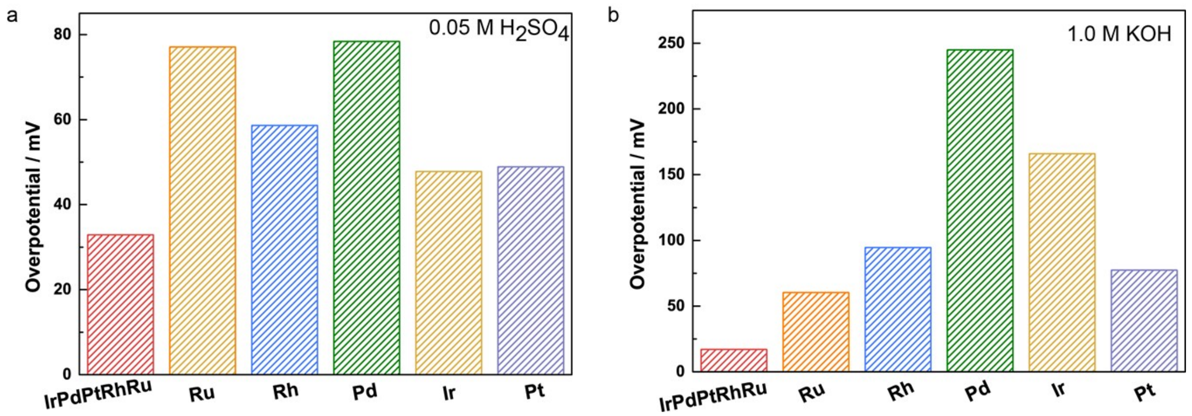

Fig. 7 Overpotential of the catalyst to achieve a geometric current density of 10 mV cm^{-2} in (a) 0.05 M H_2SO_4 and (b) 1.0 M KOH solution.

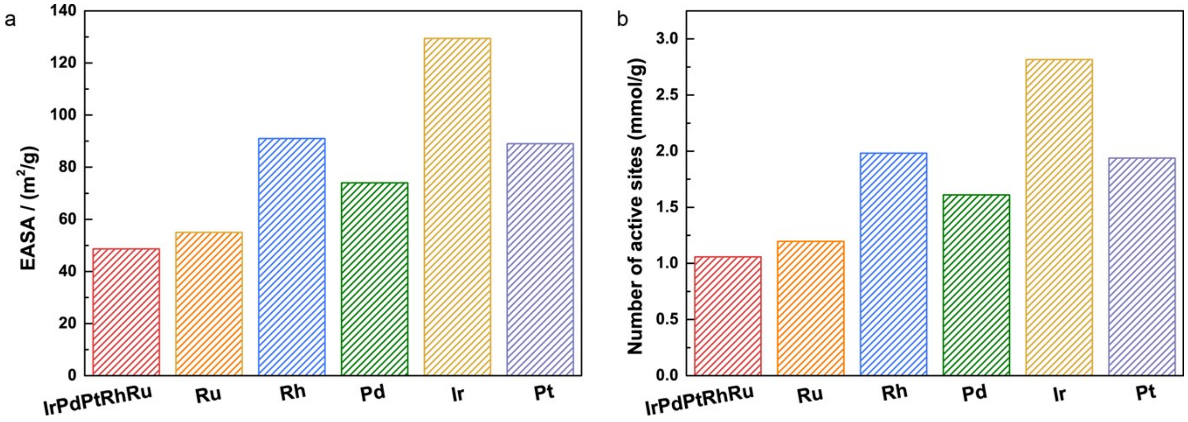

Fig. S8 (a) Specific EASA and (b) number of the active sites of the catalysts.

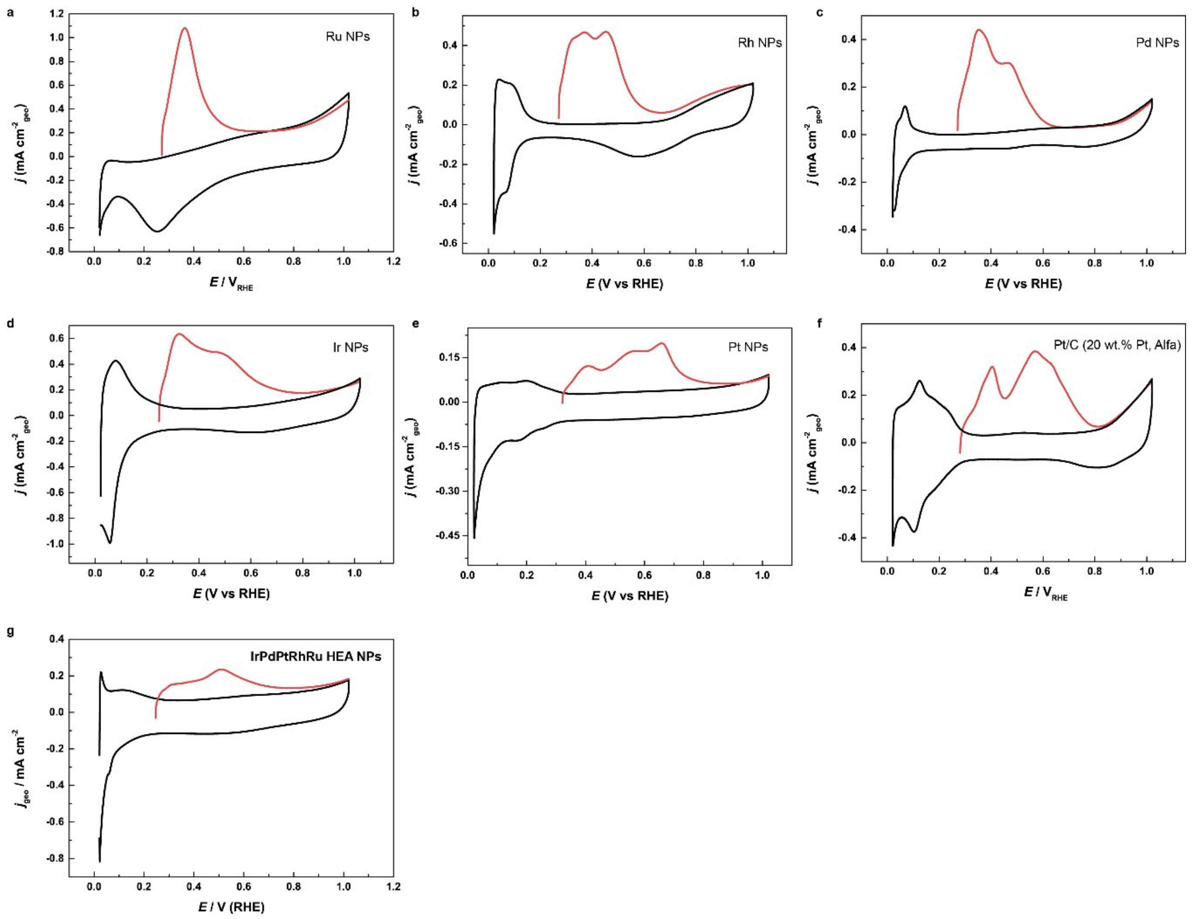

Fig. S9 Cu UPD measurement for (a) Ru, (b) Rh, (c) Pd, (d) Ir, (e) Pt, (f) commercial Pt/C, and (g) **IrPdPtRhRu HEA NPs**.

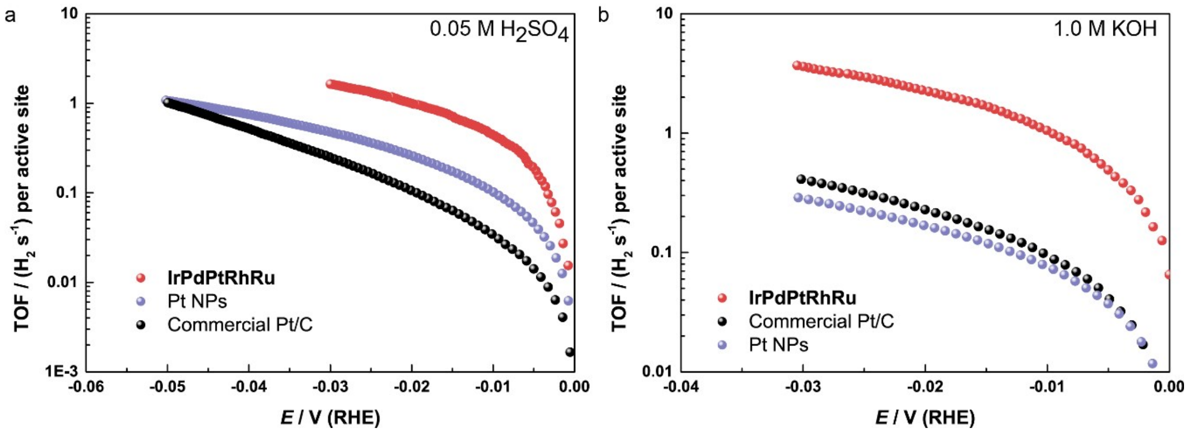

Fig. S10 Comparison of the TOF between Pt NPs, commercial Pt/C, and **IrPdPtRhRu HEA NPs** in (a) 0.05 M H_2SO_4 and (b)1.0 M KOH solution.

Table S1. The atomic percentage of each element in **IrPdPtRhRu HEA NPs** obtained by XRF and XPS.

	**Ru**	**Rh**	**Pd**	**Ir**	**Pt**
**XRF**[1]	20.75 ± 0.30	19.93 ± 0.13	20.74 ± 0.31	19.06 ± 0.40	19.51 ± 0.29
**XPS**[2]	22.67 ± 3.0	20.71 ± 2.9	21.67 ± 2.4	17.51 ± 3.0	17.40 ± 5.4

[1] the standard deviation is obtained by three independent measurements.

[2] the standard deviation comes from the uncertainties of the XPS measurement.

Table S2. The *d*-band centre of the **IrPdPtRhRu HEA NPs** and monometallic NPs.

	**Ru**	**Rh**	**Pd**	**Ir**	**Pt**	**IrPdPtRhRu**
***d*-band centre (eV)**	–4.01	–3.85	–3.48	–4.21	–4.05	–4.11

Reference

1 M. Łukaszewski, M. Soszko and A. Czerwiński, *Int. J. Electrochem. Sci.*, 2016, **11**, 4442–4469.

2 M. Quiroz, Y. Meas, E. Lamy-Pitara and J. Barbier, *J. Electroanal. Chem. Interfacial Electrochem.* 1983, **157**, 165–174.

3 C. Green and A. Kucernak. *J. Phys. Chem. B*, 2002, **106**, 1036–1047.

4 J. Mahmood, F. Li, S. Jung, M. Okyay, Ahmad, S. Kim, N. Park, H. Jeong and J. Baek, *Nat. Nanotech.*, 2017, **12**, 441–446.
